# Population Pharmacokinetics/Pharmacodynamics of Dabrafenib Plus Trametinib in Patients with BRAF-Mutated Metastatic Melanoma

**DOI:** 10.3390/cancers12040931

**Published:** 2020-04-09

**Authors:** David Balakirouchenane, Sarah Guégan, Chantal Csajka, Anne Jouinot, Valentine Heidelberger, Alicja Puszkiel, Ouidad Zehou, Nihel Khoudour, Perrine Courlet, Nora Kramkimel, Coralie Lheure, Nathalie Franck, Olivier Huillard, Jennifer Arrondeau, Michel Vidal, Francois Goldwasser, Eve Maubec, Nicolas Dupin, Selim Aractingi, Monia Guidi, Benoit Blanchet

**Affiliations:** 1 Department of Pharmacokinetics and Pharmacochemistry, Cochin Hospital, AP-HP, CARPEM, 75014 Paris, France; davidbalak3@gmail.com (D.B.); alicjapuszkiel@gmail.com (A.P.); nihel.khoudour@aphp.fr (N.K.); michel.vidal@aphp.fr (M.V.); 2 UMR8038 CNRS, U1268 INSERM, Faculty of Pharmacy, University of Paris, PRES Sorbonne Paris Cité, CARPEM, 75006 Paris, France; 3 Department of Dermatology, Cochin Hospital AP-HP, 75014 Paris, France; sarah.guegan.bart@gmail.com (S.G.); nora.kramkimel@aphp.fr (N.K.); coralie.lheure@gmail.com (C.L.); nathalie.franck@aphp.fr (N.F.); nicolas.dupin@aphp.fr (N.D.); selim.aractingi@gmail.com (S.A.); 4 Cochin Institute, INSERM U1016, University of Paris, 75014 Paris, France; anne.jouinot@aphp.fr; 5 Center for Research and Innovation in Clinical Pharmaceutical Sciences, Lausanne University Hospital and University of Lausanne, 1011 Lausanne, Switzerland; chantal.csajka@chuv.ch (C.C.); Monia.Guidi@chuv.ch (M.G.); 6 Institute of Pharmaceutical Sciences of Western Switzerland, University of Geneva, 1211 Geneva, Switzerland; 7 School of Pharmaceutical Sciences, University of Geneva, 1211 Geneva, Switzerland; 8 Department of Dermatology, Avicenne Hospital AP-HP, 93000 Bobigny, France; valentine.heidelberger@aphp.fr (V.H.); eve.maubec@aphp.fr (E.M.); 9 Department of Dermatology, Henri Mondor Hospital AP-HP, 94010 Créteil, France; ouidad.zehou@aphp.fr; 10 Service of Clinical Pharmacology, Lausanne University Hospital and University of Lausanne, 1011 Lausanne, Switzerland; Perrine.Courlet@chuv.ch; 11 Department of Medical Oncology, Cochin Hospital AP-HP, 75014 Paris, France; olivier.huillard@aphp.fr (O.H.); jennifer.arrondeau@aphp.fr (J.A.); francois.goldwasser@aphp.fr (F.G.)

**Keywords:** melanoma, BRAF, dabrafenib, hydroxy-dabrafenib, trametinib, population pharmacokinetics, pharmacodynamics

## Abstract

Patients treated with dabrafenib/trametinib (DAB/TRA) exhibit a large interindividual variability in clinical outcomes. The aims of this study were to characterize the pharmacokinetics of DAB, hydroxy-dabrafenib (OHD), and TRA in BRAF-mutated patients and to investigate the exposure–response relationship for toxicity and efficacy in metastatic melanoma (MM) patients. Univariate Fisher and Wilcoxon models including drug systemic exposure (area under the plasma concentration curve, AUC) were used to identify prognostic factors for the onset of dose-limiting toxicities (DLT), and Cox models for overall (OS) and progression-free survival (PFS). Seventy-three BRAF-mutated patients were included in pharmacokinetic (n = 424, NONMEM) and 52 in pharmacokinetic/pharmacodynamic analyses. Age and sex were identified as determinants of DAB and OHD clearances (*p* < 0.01). MM patients experiencing DLT were overexposed to DAB compared to patients without DLT (AUC: 9624 vs. 7485 ng∙h/mL, respectively, *p* < 0.01). Eastern Cooperative Oncology Group Performance Status (ECOG PS) ≥ 2 and plasma ratio AUC_OHD_/AUC_DAB_ ≥ 1 were independently associated with shorter OS (HR: 6.58 (1.29–33.56); *p* = 0.023 and 10.61 (2.34–48.15), *p* = 0.022, respectively). A number of metastatic sites ≥3 and cerebral metastases were associated with shorter PFS (HR = 3.25 (1.11–9.50); *p* = 0.032 and HR = 1.23 (1.35–10.39), *p* = 0.011; respectively). TRA plasma exposure was neither associated with toxicity nor efficacy. Our results suggest that early drug monitoring could be helpful to prevent the onset of DLT in MM patients, especially in fragile patients such as the elderly. Regarding efficacy, the clinical benefit to monitor plasma ratio AUC_OHD_/AUC_DAB_ deserves more investigation in a larger cohort of MM patients.

## 1. Introduction

Mutations in the RAS-RAF-MEK-ERK signaling pathway, a kinase cascade that regulates cellular growth and survival [1], have been reported in different types of cancers. BRAF is a serine/threonine kinase that catalyzes the phosphorylation and activation of MEK1 and MEK2. BRAF is reportedly mutated in 3–5% of non-small-cell lung cancer (NSCLC), 10% of colorectal cancers, 10–70% of thyroid cancers, and 52% of cutaneous melanomas [2]. Dabrafenib (DAB), a reversible ATP-competitive inhibitor, selectively inhibits BRAF^V600E/K^-mutant proteins [3]. In patients with BRAF^V600^ metastatic melanoma (MM), DAB in combination with trametinib (TRA), a MEK inhibitor, has demonstrated higher response rate and improved survival compared with DAB monotherapy [4]. Thus, the combination of DAB plus TRA (CombiDT) led to a long-term benefit with a survival rate of 34% at five years [4]. However, 18% of patients permanently discontinued DAB or TRA because of severe adverse events (AE), the most common being pyrexia.

The oral route of DAB and TRA has improved patient’s quality of life in comparison with chemotherapy such as dacarbazine. However, patients in “real-life” cohorts exhibit a large interindividual variability (IIV) in clinical outcomes, such as toxicity [5] and efficacy [6], which can result in dose-limiting toxicities (DLT) or early disease progression. As previously reported with vemurafenib (BRAF inhibitor), variability in drug pharmacokinetics can contribute to early progression in BRAF-mutated MM patients [7,8,9,10]. To date, no study has shown an exposure–response relationship for efficacy of DAB. Regarding safety, Menzies et al. reported that the onset of pyrexia is associated with an increased plasma DAB exposure [11], but a recent study did not confirm this result [12]. Nevertheless, DLTs seem more frequent in patients with higher plasma DAB exposure [5]. DAB undergoes oxidative metabolism via cytochrome P450 (CYP) 3A4 and CYP2C8 to form an active metabolite, hydroxy-dabrafenib (OHD) which has a twofold higher potency as an inhibitor of mutant BRAF [13]. Furthermore, the mean OHD-to-DAB exposure ratio in plasma is 0.8. These data suggest a significant pharmacodynamic contribution of OHD in BRAF-mutated patients treated with DAB. However, the influence of OHD pharmacokinetics on both toxicity and efficacy was rarely studied in previous researches [11,12], as OHD compound is not marketed. In BRAF-mutated MM patients treated with TRA monotherapy vs. CombiDT, a phase 2 trial showed that trough plasma concentration of TRA above 10.6 ng/mL was associated with a better clinical efficacy [14,15], but this result was not confirmed in a phase 3 [14,16] trial. Taken together, these data suggest that the exposure–response relationship for toxicity and efficacy should be further investigated in BRAF-mutated MM patients treated with CombiDT, especially in routine clinical settings.

Finally, BRAF and MEK inhibitors exhibit a moderate-to-large IIV in their pharmacokinetics [17]. Identifying the factors responsible of their variabilities is mandatory for patient care because they have a narrow therapeutic index and are often toxic at therapeutic dose. As far as we know, population pharmacokinetic (popPK) analyses for DAB [18] and TRA [14] were only conducted within selected patient populations from clinical trials. However, “real life” patients are more fragile than those enrolled in randomized clinical trials for different reasons, e.g., general condition, comorbidities, and polymedication. Therefore, a larger IIV in pharmacokinetics can be expected in routine clinical settings and should be explored.

The aims of this study were to characterize the pharmacokinetic variability of DAB, OHD, and TRA in a “real life” cohort of BRAF-mutated patients, then to investigate a potential exposure–response relationship for toxicity and efficacy in BRAF-mutated MM patients treated with DAB plus TRA.

## 2. Results

### 2.1. Pharmacokinetic Data

Seventy-three patients treated with DAB were included in this study. Among these 73 patients, 60 patients (82%) were concomitantly treated with TRA. Table 1 presents baseline characteristics of the two populations used for the building of pharmacokinetic models for DAB/OHD and TRA, as well as those of 52 MM patients included in the PK/PD analyses. A total of 424 and 318 concentrations were used for the model development of DAB/OHD and TRA, respectively.

### 2.2. Pharmacokinetic Data

#### 2.2.1. Dabrafenib/Hydroxy-dabrafenib Model

**Figure 1:**
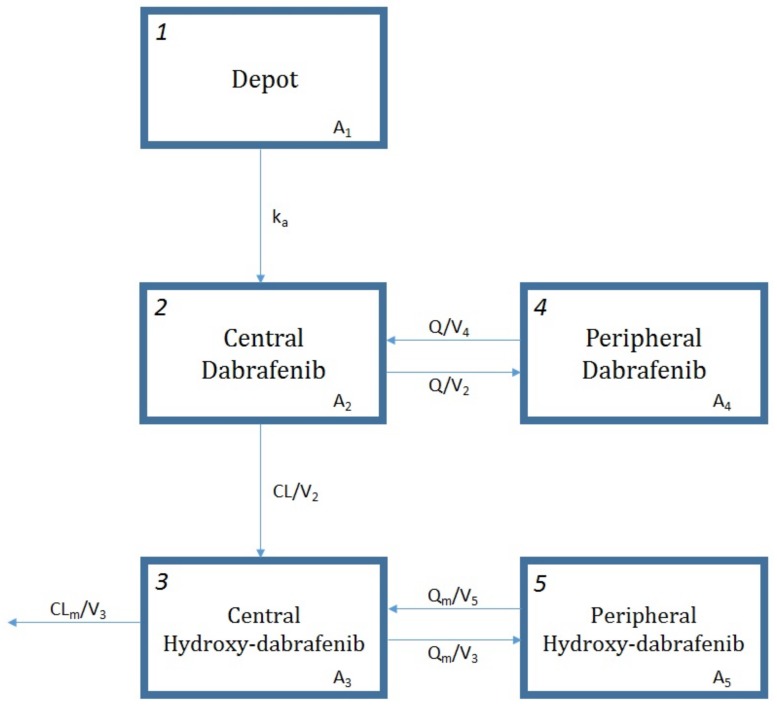
Scheme of the pharmacokinetic model for dabrafenib and hydroxy-dabrafenib. A_1_, A_2_, and A_4_ are the amounts of dabrafenib (DAB) in gastrointestinal tract, central compartment, and peripheral compartment, respectively. A_3_ and A_5_ are the amounts of hydroxy-dabrafenib (OHD) in central compartment and peripheral compartment, respectively. k_a_ represents the first-order absorption rate constant, CL/V_2_ the first-order metabolic rate constant, CL_m_/V_3_ the first-order OHD elimination rate constant, Q/V_4_ and Q/V_2_ the first-order DAB distribution and redistribution rate constants, and Q_m_/V_5_ and Q_m_/V_3_ the first-order OHD distribution and redistribution rate constants. CL/F and CL_m_/F are DAB and OHD clearances; Q/F and Q_m_/F are DAB and OHD intercompartmental clearances; V_2_ and V_3_ are DAB and OHD central volumes of distribution; V_4_and V_5_ are DAB and OHD peripheral volumes of distribution.

DAB and OHD data were best described with a two-compartment model for each molecule (Figure 1), drug exclusive elimination via irreversible conversion to OHD and first-order elimination of metabolite (ΔOFV = −24.306, *p* < 0.01 with respect to a one compartment model for both). A first-order absorption with a lag time adequately described the absorption phase (ΔOFV = −52.344, *p* < 0.01 with respect to the model with only k_a_). The addition of an IIV on DAB apparent central volume of distribution (V_2_/F), on OHD apparent clearance (CL_m_/F) and central volume of distribution (V_3_/F) resulted in a significant decrease in OFV compared to IIV on DAB apparent clearance (CL/F) alone (ΔOFV = −309.831, *p* < 0.01). Proportional errors were selected to describe the RUV of both DAB and OHD concentrations. The correlation between DAB and OHD concentrations improved markedly data description (ΔOFV = −353.241, *p* < 0.01). Moreover, the inter-occasion variability (IOV) on CL/F is estimated to be 17% (ΔOFV = −52.291, *p* < 0.01).

DAB and OHD pharmacokinetics did not differ in patients treated with DAB monotherapy vs. combiDT (ΔOFV = −2.9, *p* > 0.05) or concomitant proton-pump inhibitor (PPI) intake (ΔOFV = −3.5, *p* > 0.05). Among all the tested covariates, age was identified as an influential covariate for both CL/F and CL_m_/F (ΔOFV < −10.613, *p* < 0.01) and sex for CL/F (ΔOFV = −8.039, *p* < 0.01). CL/F is reduced by 17% in women vs. men and by 55% when comparing 20-year-old to 90-year-old patients. A similar decrease (51%) is observed in CL_m_/F under the same age variation. These pharmacokinetic parameter-covariate relationships were all retained in the final model after multivariate combination and backward deletion step.

**Figure 2:**
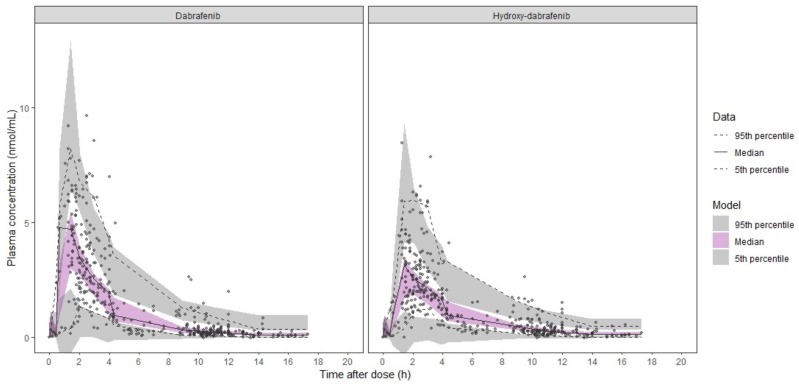
Prediction-corrected visual predictive check of dabrafenib/hydroxy-dabrafenib final model. Dots, continuous, and dashed lines represent DAB (left panel) and OHD (right panel) prediction-corrected plasma concentrations with median and 90% percentile intervals, respectively. Shaded areas represent the 90% confidential intervals around the model-based 5%, 50%, and 95% percentiles.

The base and final models’ parameter estimates as well as their bootstrap estimations are presented in Table 2. The model was judged reliable since all bootstrap median parameter estimates values are contained within the bootstrap 95% percentile interval (PI_95%_) and differ by less than 10% from the population parameter estimates, exception made for the IIV on V_2_/F and V_3_/F. The diagnostic plots (Figures S1 and S2) and pcVPC (Figure 2) support the good predictive performances of the model.

**Figure 3:**
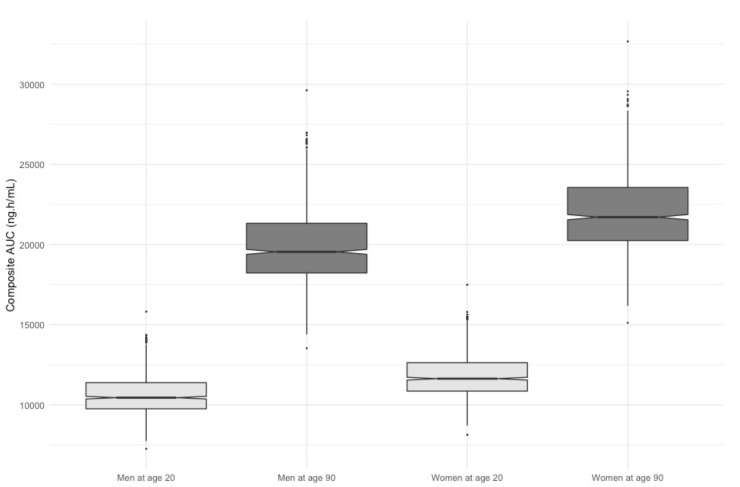
Simulations of composite AUC for men and women at age 20 versus age 90.

Figure 3 assesses the clinical relevance of the retained covariates presenting the simulated composite area under the plasma concentration curve (AUC) for men and women at the age of 20 and 90 treated with DAB at 150 mg twice daily. The median composite AUC were 10,447 (7254–15,813) ng∙h/mL, 19,542 (13,531–29,621) ng∙h/mL, 11,632 (8126–17,486) ng∙h/mL, 21,707 (15,117–32,666) ng∙h/mL for men at age 20, men at age 90, women at age 20, and women at age 90, respectively. These results clearly show the predominant effect of age on DAB and OHD pharmacokinetics.

#### 2.2.2. Trametinib Model

A two-compartment model with first order absorption and elimination best described TRA pharmacokinetics (ΔOFV = −12.711, *p* < 0.01 compared to a one-compartment model). The use of a lag time yielded a better fit (ΔOFV = −8.274, *p* < 0.01). Inclusion of an IIV on Q/F but not of an IOV in addition to IIV on CL/F markedly improved data description (ΔOFV = −9.723, *p* < 0.01 and ΔOFV = 0, *p* > 0.05, respectively). None of the tested covariates, including PPI intake, could explain this variability (ΔOFV < 1.426, *p* > 0.05). An additive model was selected to describe the RUV.

**Figure 4:**
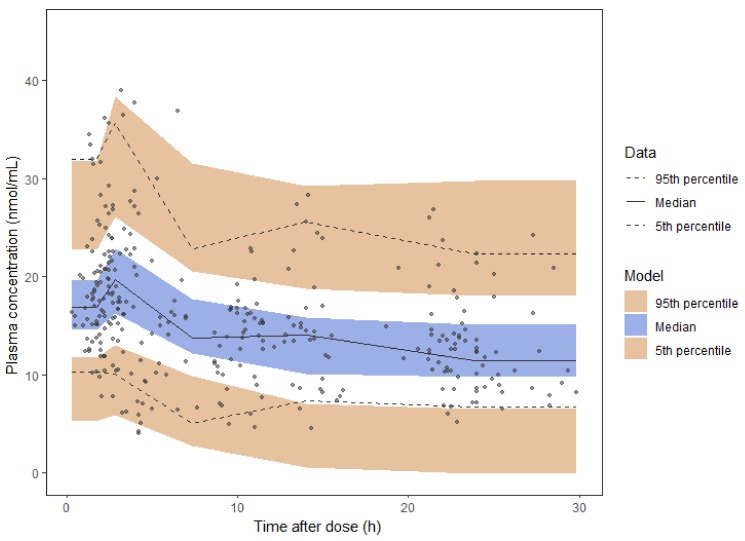
Prediction-corrected visual predictive check of trametinib final model. Dots, continuous, and dashed lines represent prediction-corrected TRA plasma concentrations with median and 90% percentile intervals, respectively. Shaded areas represent the 90% confidential intervals around the model-based 5%, 50%, and 95% percentiles.

Table 3 presents the final TRA pharmacokinetic model together with the bootstrap results. The model was judged reliable since all bootstrap median parameter estimates values are contained within the PI_95%_ and differ by less than 13% from the population parameter estimates. The diagnostic goodness-of-fit plots (Figure S3) and pcVPC (Figure 4) further supported model adequacy. Moreover, the external model validation showed a non-significant bias (mean prediction error, MPE) of 2% (CI_95%_: −4–7%) at the individual level.

### 2.3. Exposure-Toxicity Relationship

**Figure 5:**
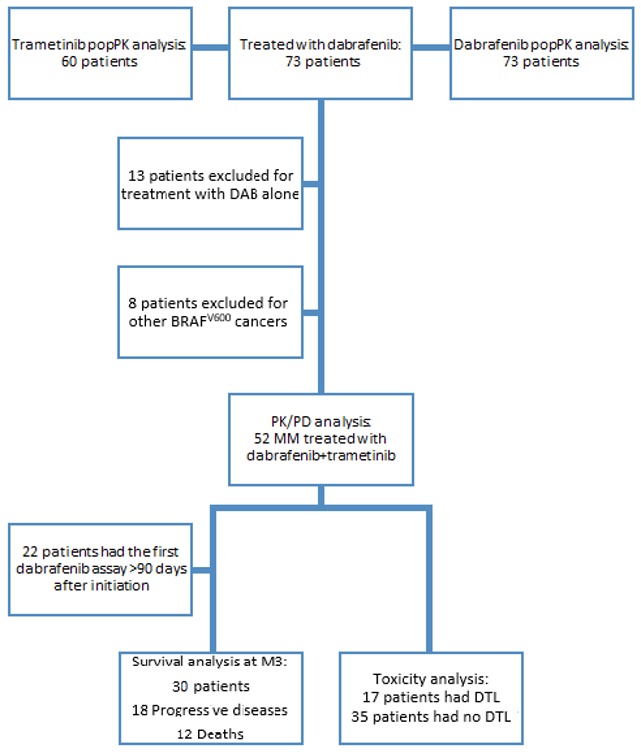
Study flowchart.

The PK/PD study was conducted in 52 MM patients harboring BRAF^V600^ mutation and concomitantly treated with DAB and TRA (Figure 5). Among them, three patients (6%) started CombiDT at reduced dosing (75 mg twice daily for DAB and 1 mg daily for TRA) because of extreme age (*n* = 2; 89 and 90 years old) and previous vemurafenib/cobimetinib-induced hepatitis (*n* = 1). The median duration of DAB treatment was 9.9 months (1.1–93.6). The median follow-up duration was 13.7 months (4.0–95.1). At data cut-off in May 2019, 22 patients (42%) were still treated with DAB.

Of 52 patients, 34 (65%) patients experienced AE, 12 patients (23%) experienced a DAB-related DLT, and 17 (33%) a TRA-related DLT with a median time to onset of 110 days (13–344) and 104 days (13–368), respectively. The most common AE were fatigue (12 cases, 23%), creatine phosphokinase increase (12 cases, 23%), pyrexia (10 cases, 19%), rash (5 cases, 10%), liver enzymes elevation (5 cases, 10%), sarcoid-like reactions (5 cases, 10%), diarrhea (4 cases, 8%), nausea (3 cases, 6%), arthralgia (3 cases, 6%), epistaxis (3 cases, 6%). Nine (17%) patients displayed common terminology criteria (CTC) grade 3 or 4 AE. DLTs included 1 case of grade 4 pancreatitis, 2 cases of grade 3 neutropenia, and 1 case each of grade 3 AE between creatine phosphokinase increase, pyrexia, panniculitis, interstitial lung disease, cardiac disorder, and lymphoedema. DAB was discontinued permanently in 4 patients (8%) and reduced in 8 patients (15%). TRA was discontinued permanently in 5 patients (10%) and reduced in 12 patients (23%). The last AUC_DAB_ in patients who experienced DLT was statistically higher than the average AUC_DAB_ over treatment course in patients without DLT (9624 ng∙h/mL vs. 7485 ng∙h/mL, respectively; *p* < 0.01) (Table 4). All severe adverse events were tolerable for patients after dosing reduction regardless of drug (DAB, TRA). A 1.8-fold decrease in mean AUC_DAB_ was observed after DAB dose reduction (9014 vs. 5037ng∙h/mL, respectively, paired *t*-test student, *p* < 0.001, *n* = 8). By contrast, no relationship was found between baseline parameters such as age, BMI, sex, Eastern Cooperative Oncology Group Performance Status (ECOG PS), and the onset of DLT. Regarding patients who experienced TRA-related DLT, no relationship was found neither between the last AUC_TRA_ nor with the baseline parameters (Table 4).

### 2.4. Exposure-Survival Relationship

Twenty-two patients were excluded from the analysis of exposure-survival relationships because the first plasma assessment was done after more than three months after DAB initiation (Figure 5). Among the 30 included patients, the median PFS and OS were 7.0 months (2.0–95.1) and 13.4 months (4.0–95.1), respectively. The median blood samples per patient during the first three months of treatment were 2 (1–3). The median AUC_M3,DAB_, AUC_M3,OHD_, and AUC_M3,TRA_ were 7722 ng∙h/mL (3656–11054), 6082 ng∙h/mL (3651–9113), and 292 ng∙h/mL (111–750), respectively.

In univariate analysis, increased AUC_M3,OHD_, AUC_M3,OHD/DAB_ ≥1, and ECOG PS ≥2 were statistically associated with OS. In multivariate analysis, AUC_M3,OHD/DAB_ ≥ 1 and AUC_M3,OHD_ were still independently associated with shorter OS (10.61 (2.34–48.15); *p* = 0.022 and HR = 1.61 (1.07–2.45); *p* = 0.023, respectively) as well as ECOG PS ≥ 2. Moreover, in univariate analysis, increased AUC_M3,OHD_, a number of metastatic sites ≥ 3, and the presence of cerebral metastases were statistically associated with shorter PFS. Finally, in multivariate analysis, a number of metastasis site and cerebral metastases were still associated with shorter PFS (HR = 3.25 (1.11–9.50); *p* = 0.032 and HR = 1.23 (1.35–10.39); *p* = 0.011, respectively) (Table 5).

## 3. Discussion

The DAB/OHD model described in the present analysis is the first ever published to describe DAB and OHD data simultaneously on a real-life cohort. Estimated CL/F, Q/F, and lag time are close to the values of 17.0 L/h, 3.30 L/h, and 0.482 h, respectively, reported in reference models built upon clinical trial data [12,18], respectively. Clearance auto-induction could not be modeled in this study since all subjects were sampled at steady state, and hard capsule is the only formulation available in France for oral administration of DAB. The estimated central and peripheral volumes of distribution are however markedly lower. Important IIV characterizes DAB/OHD pharmacokinetics even after covariate integration. Our analysis confirms the contribution of sex on CL/F found in the reference model [18], but not of bodyweight, which might be due to its different distribution between the two study populations. Aside from sex, the key covariate for DAB and OHD clearances was age; these covariates explained 47% and 20%, respectively, of their base model variances. Age was, however, not retained in the reference model [18]. We hypothesize that selected patients enrolled in clinical trials are younger than patients in “real-life” cohorts; indeed, our cohort is slightly older (median age of 53 vs. 61 years). Since DAB is metabolized by CYP3A4 and CYP2C8, it is expected to be a victim of drug–drug interactions. Unfortunately, none of the patients included in this study were treated with a strong inducer or inhibitor of CYP3A4 or CYP2C8, and their impact on DAB/OHD pharmacokinetics could not be investigated thoroughly.

The TRA model developed upon this “real-world” cohort is a two-compartment model with parameter estimates similar to the reference model from the literature [14]. None of the available covariates could explain the substantial IIV on clearance. Of note and as previously reported, TRA pharmacokinetics is not subject to large variation over time.

In daily clinical practice, the use of PPI is a concern in cancer patients treated with tyrosine kinase inhibitors (TKI) [19]. Indeed, the increase in gastric pH induced by PPI can significantly decrease the bioavailability of some TKIs such as pazopanib and erlotinib and therefore result in lesser efficacy of these TKIs [20,21,22]. The present study suggests that the concomitant intake of PPI with DAB or TRA does not significantly contribute to the variability in their pharmacokinetics. This result is in agreement with manufacturer product information [23,24]. Furthermore, the univariate Cox analysis did not identify the concomitant use of PPI as a risk factor of shorter survival, which supports the fact that the concomitant use of PPI is not clinically meaningful in patients treated with CombiDT.

In the present study, the frequency of dose reduction related to CombiDT is quite similar to that reported in the phase 3 trial (33%) [25]. Interestingly, the last AUC_DAB_ preceding the DAB-related DLT onset is statistically higher than AUC_DAB_ of patients who did not experience any DLT. Similarly, Rousset et al. reported an association between a high trough DAB concentration and the onset of DLT in 27 MM patients [5]. By contrast, Kim et al. showed that neither dabrafenib AUC nor trough concentration are associated with incidence of treatment interruption [12]. The discrepancy between the result from Kim et al. and ours is in part methodological since we chose DLT (including dose reduction) as clinical endpoint to conduct PK/PD analysis. Rousset et al. have also established a plasma through threshold of 48 ng/mL as the concentration predicting the occurrence of DLT [5]. In the present study, we could not confirm this result, since we preferred using AUC for PK/PD analysis because it better reflects the daily exposure to the drug than trough concentration. Furthermore, blood samples are often drawn at any time during two dose intakes and not at rigorous through concentration in daily clinical practice. In this context, our Bayesian approach of model-estimated AUC should be more convenient to perform plasma monitoring of dabrafenib.

To date, there is no reliable biomarker to identify responder patients to CombiDT [26]. Therefore, the clinical benefit assessment of this targeted combination therapy largely relies on clinical and radiological criteria in BRAF-mutated MM patients. In the present study, ECOG PS ≥2, a number of metastatic sites ≥3, and presence of cerebral metastases are independent predictors of shorter OS and shorter PFS, respectively. These parameters are well documented as prognostic factors of survival [27,28,29]. Surprisingly, this study highlights that a high AUC_M3,OHD_ is associated with a shorter OS. The median AUC_OHD_/AUC_DAB_ ratio is of 0.80 (0.50–1.32) in our cohort, which is in agreement with the expected value [13]. Interestingly, when adjusted to ECOG PS, the AUC_M3,OHD_/AUC_M3,DAB_ ratio ≥1 (highest quartile of the AUC_M3,OHD_/AUC_M3,DAB_ ratio distribution) is also an independent predictor of shorter OS (HR=10.61 (2.34–48.15), *p* = 0.022), which suggests that patients metabolizing DAB to OHD quickly might be at risk of early death. This result is surprising because an in vitro study reported a two-fold higher pharmacological potency of OHD compared to DAB in regard with BRAF inhibition [13]. However, preclinical data suggest that only DAB and desmethyl-dabrafenib may cross intact blood brain barrier [23]. In the present cohort, 72% of patients discontinued DAB treatment because of cerebral progression. Interestingly, the AUC_M3,OHD_/AUC_M3,DAB_ ratio was statistically higher in patients experiencing cerebral progression compared to those with extra-cerebral progression (0.9 (0.5–1.3) vs. 0.8 (0.6–0.8), Wilcoxon test *p* = 0.0077). Thus, all patients experiencing extra-cerebral progression exhibited AUC_M3,OHD_/AUC_M3,DAB_ ratio ≤ 0.8. Taken together, these results indicate that the antitumoral effect of DAB therapy on brain metastases is limited in patients with high AUC_M3,OHD_/AUC_M3,DAB_ ratio because of the lack of OHD diffusion into the brain. This observation could explain in part the shorter OS in patients with high AUC_M3,OHD_.

Targeted therapies including kinase inhibitors frequently present different off-targets, as previously demonstrated for sunitinib and its active metabolite by kinomic analysis [30]. Miao et al. have shown that vemurafenib and DAB have a different impact on the expression of protein kinases [31]. Furthermore, several resistance mechanisms to BRAF inhibitors have been identified: dimerization of aberrantly spliced BRAF, amplification of mutant BRAF, acquisition of mutations in RAS or MEK, upregulation of MAP3K8/COT, loss of NF1, upregulation of the EGF receptor-SRC family kinase-STAT3 signaling pathway, and PI3K–PTEN–AKT pathway upregulating mutations [32]. Taken together, these data show the complexity of pharmacodynamic effect of BRAF inhibitors. In this context, DAB and OHD could differently affect the reprogramming of the human kinome and ultimately bypass the targeted inhibition [33]. One cannot exclude that high concentration of OHD could have deleterious pharmacodynamic effect on some kinases, resulting in less efficacy of DAB therapy in our cohort. Further research is warranted to decipher this point. Overall, the association between high AUC_M3,OHD_ and shorter OS should be interpreted with caution because of the small size of our cohort (*n* = 30 MM patients in multivariate Cox analysis). Further investigations in a larger cohort of MM patients are required to confirm this result.

The delayed onset of DAB-related DLT indicates that plasma DAB monitoring should benefit MM patients after a 3 month time frame of treatment. Furthermore, early plasma DAB monitoring could be useful to prevent the onset of DLT in elderly patients who are at risk of plasma overexposure because of a decreased DAB metabolic clearance. In this context, a reduced starting dose of DAB associated with a pharmacokinetic-guided strategy could be proposed to prevent early DLT in elderly patients. Regarding TRA, we did not find any relationship between plasma drug exposure and clinical endpoints (survival, DLT), as previously reported [5,12]. This result supports the lack of clinical benefit in monitoring TRA concentration in daily clinical practice, except to ensure patients adherence to treatment.

## 4. Materials and Methods

### 4.1. Ethics

This study is in accordance with the 2008 declaration of Helsinki and was approved by the local ethics committee in oncology (N° CLEC 211218ACBB1). All patients provided written informed consent for the collection of their medical data.

### 4.2. Study Population

Seventy-three outpatients treated with DAB were enrolled from July 2015 to June 2017 in this observational multicentric study including three hospitals of the Assistance Publique—Hôpitaux de Paris (Cochin, Henri Mondor, and Avicenne). To be eligible, patients needed to meet the following criteria: a minimal age of 18 years, metastatic BRAF^V600^-mutated solid tumors, treatment with DAB or CombiDT with at least one available DAB concentration measurement.

Patients initiated DAB at the recommended daily dose of 150 mg twice daily. In patients treated with CombiDT, TRA was started at 2 mg once daily. Reduced starting doses were allowed at physician discretion based on comorbidities and patients’ general condition. All AE were graded using the National Cancer Institute Common Toxicity Criteria v4.0. DAB treatment was continued until disease progression, unacceptable toxicity, or unfitness to receive it.

### 4.3. Analytical Method

DAB, dabrafenib-d9 (internal standard, IS), and TRA were purchased from LGC Standards (Molsheim, France). A custom synthesis was developed by Shanghai Medicilon (Shanghai, China) for OHD. The plasma concentrations were assayed using a validated high-performance liquid chromatography-tandem mass spectrometry (HPLC-MS/MS) (ATSQ Quantum Ultra^®^mass spectrometer, ThermoFisher, Les Ulis, France). Chromatographic separation was performed on Accucore^®^ C18 (2.1 × 50 mm; 2.6 µm) analytical column (ThermoFisher Scientific, Les Ulis, France) associated with a guard column packed with the same bonded phase. Two hundred microliters (µL) aliquot of plasma (calibration standard, internal quality control or patient sample) was mixed with 20 µL of IS solution. Then 600 µL acetonitrile with 1% (*v*/*v*) formic acid was added. Samples were mixed and centrifuged, and the supernatant was evaporated and recovered with 200 µL of mobile phase: water/methanol (70:30%, *v*/*v*) with formic acid (0.1% *v*/*v*). Ten microliters of this mixture were injected into the HPLC-MS/MS system. The calibration standards ranged from 10 to 2000 ng/mL for DAB and OHD and from 5 to 50 ng/mL for TRA.

The precision and accuracy of the method were determined with low-, medium-, and high-quality control (QC) samples at 25, 200, and 1500 ng/mL for DAB and OHD, and 8, 15, and 40 ng/mL for TRA. The inter-assay precisions means were satisfactory, with coefficients of variation within 1.59% to 14.97%. The deviations between nominal and measured concentrations of the QCs (% bias) were comprised between −1.19% and 10.93%. This method was therefore reproducible and reliable for research purposes.

Blood samples were collected in heparinized tubes during the treatment course, and the exact sample time was recorded. Plasma was separated by centrifugation (3000 rpm, 5 min), and stored at −20 °C until analysis.

### 4.4. Population Pharmacokinetic Analysis

The non-linear mixed effects modeling program NONMEM v7.4.1 [34] with the Perl-Speaks NONMEM (PsN) Toolkit v4.8.0 [35] was used to build DAB/OHD and TRA models. Pirana v2.9.2 was used to assist in model development. Statistical analyses and graphical exploration were performed by R program v3.6.1 with RStudio v1.1.383 (http://www.r-project.org). Molar units were used for DAB/OHD analyses. Plasma samples with concentrations below the lower limit of quantification of the assay, non-reliable time information about blood sampling, or without dose information were excluded from the analysis.

#### 4.4.1. Structural and Statistical Model

A stepwise strategy was used to identify the base models that best fitted DAB/OHD and TRA pharmacokinetic data. Different structural models such as one or two compartments, with linear absorption with or without lag time, were compared. DAB and OHD were modeled simultaneously testing multi-compartment models for both compounds. We hypothesized that DAB was cleared by complete oxidation to OHD, since it is not eliminated as unchanged molecule in urine and accounts for less than 22% in feces [36]. Moreover, the first-order absorption rate constant (k_a_) of DAB was fixed to 1.8 h^−1^ according to a previously published value [18] to allow an adequate estimate of all pharmacokinetic parameters. Since DAB and TRA are administered orally, the pharmacokinetic parameters represent apparent values. Exponential errors following a log-normal distribution were assumed for the description of IIV of all the pharmacokinetic parameters. Inter-occasion variability (IOV) was tested in both DAB/OHD and TRA models to assess differences in individual parameters across study occasions. Additive, proportional, and mixed error models were compared to depict the residual unexplained variability (RUV) for both drugs and metabolite. The correlation between DAB and OHD data was tested using the L2 item in NONMEM.

#### 4.4.2. Covariate Analysis

Continuous and categorical dichotomous covariates assessed to determine their impact on the drugs pharmacokinetics were: total body weight, body mass index (BMI), free-fat mass (FFM), body surface area (BSA), age, aspartate aminotransferase (AST), alanine aminotransferase (ALT), total bilirubin, albumin, C-reactive protein (CRP), sex, intake of proton-pump inhibitors (PPI), and cotreatment with TRA in the DAB/OHD analysis. The FFM was calculated according to the formulas proposed by Janmahastian et al. [37]. Continuous covariates were centered and normalized on their median value, while categorical covariates were coded as 0/1 for absence/presence of the covariate, respectively. Information on categorical factors was complete for all the patients enrolled in the analyses. Missing values were imputed to the population median value for continuous covariates. They represented 4%, 19%, 20%, 37%, and 42% of DAB/OHD data and 5%, 17%, 17%, 35%, and 42% of TRA observations for the body weight, the transaminases, total bilirubin, albumin, and CRP, respectively. Potential and physiologically plausible relationships between pharmacokinetic parameters and patients’ characteristics were first investigated graphically. A stepwise forward insertion/backward deletion approach was then undertaken describing the effect of each covariate on a pharmacokinetic parameter by linear or allometric functions as appropriate.

#### 4.4.3. Parameter Estimation and Model Selection

DAB/OHD and TRA models were fitted using the first-order conditional estimation method with interaction (FOCEI). The log-likelihood ratio test, based on the reduction of the objective function value (ΔOFV) provided by NONMEM, was used to discriminate between nested models. An OFV decrease of 3.84 (*p* = 0.05) and an increase of 6.63 (*p* = 0.01) points was considered statistically significant for one additional parameter in the model-building process or forward insertion and backward-deletion covariate steps, respectively (ΔOFV between any two nested models approximates a χ² distribution). Goodness-of-fit plots, precision and plausibility of parameter estimates, and shrinkage were also used as evaluation criterion for model selection.

#### 4.4.4. Model Validation and Assessment

The stability and precision of the final DAB/OHD and TRA population pharmacokinetic model were assessed by PsN-Toolkit’s bootstrap method. Median parameter estimates were compared with median values derived from 500 replicates of the initial datasets. For each final model, a prediction-corrected visual predictive check (pcVPC) was also performed by using PsN-Toolkit and the R packages “vpc” [38] and “nonmem2R” [39].

The final TRA model was externally validated using 46 additional samples collected from an independent group of 15 patients treated with TRA followed routinely at the University Hospital of Lausanne. TRA plasma concentrations were predicted by post hoc Bayesian forecasting (option MAXEVAL = 0 in NONMEM). The predictive performance of the model was assessed in terms of bias (mean prediction error, MPE) and precision (root mean square prediction error, RMSE) [40]. External validation of DAB/OHD could not be performed due to the lack of an independent set of patients with available drug and metabolite concentrations.

The clinical relevance of the retained covariates impacting the pharmacokinetics of DAB and OHD was assessed by performing simulations of 1000 patients. Model-predicted composite AUC calculated as AUC_DAB_+AUC_OHD_, with AUC_DAB_ and AUC_OHD_, respectively, estimated by classic equations, were compared for different influential characteristics.

### 4.5. Pharmacokinetic/Pharmacodynamic Analysis

Pharmacokinetic/pharmacodynamic analysis was conducted in BRAF^V600^-mutated MM patients concomitantly treated with CombiDT. Regarding safety, the onset of DLT was considered as the clinical endpoint. A DLT was defined as any toxicity leading to dose reduction or temporary or permanent discontinuation of treatment. Regarding survival, the primary end point was progression free survival (PFS), the time from DAB and TRA treatment initiation to documented progression event (either clinical or radiological progression) or death from any cause. The secondary end-point was overall survival (OS), defined as the time from DAB and TRA treatment initiation to death from any cause. Radiographic evidence of progression was defined according to modified Response Evaluation Criteria in Solid Tumor (RECIST) v1.1 [41].

### 4.6. Statistical Analysis for Survival and Toxicity

Systemic exposures to DAB, OHD, and TRA (AUC_DAB_, AUC_OHD_, and AUC_TRA_, respectively) were calculated with classic equations, composite DAB and OHD AUC (AUC_DAB+OHD_) as previously explained and AUC_OHD/DAB_ as the ratio of AUC_OHD_ over AUC_DAB_. Univariate Fisher and Wilcoxon models were performed to identify which parameters could contribute to the onset of DLT. The following variables were tested: sex, age at treatment initiation, BMI, Eastern Cooperative Oncology Group Performance Status (ECOG PS: grade 0-1 vs ≥2), lactate dehydrogenase (LDH) level (<1.5N vs. ≥1.5N), AUC_DAB_, AUD_OHD_, AUC_DAB+OHD_, and AUC_TRA_. The last AUC before the onset of DLT was compared with the average AUC estimated over the treatment course in patients who did not experience any DLT.

For the analysis of exposure–survival relationships, the following variables were tested for their influence on PFS and OS using univariate Cox proportional hazards models: sex, age at treatment initiation, BMI, ECOG PS (grade ≥ 2 vs. 0–1), cerebral metastases (presence vs. absence), a number of metastatic sites (≥3 vs. <3), LDH level (≥1.5N vs. <1.5N ), PPI intake (no intake vs. intake), and mean estimated AUC during the first three months of treatment for DAB (AUC_M3,DAB_), OHD (AUC_M3,OHD_), composite (AUC_M3,DAB+OHD_), ratio (AUC_M3,OHD/DAB_), and TRA (AUC_M3,TRA_). Multivariate Cox models according to a stepwise procedure were tested including all variables which were significant at 5% level in the univariate analyses. All the pharmacokinetic/pharmacodynamic analyses were performed using R program v3.6.1 with RStudio v1.1.383 (http://www.r-project.org).

## 5. Conclusions

In conclusion, this study presents two validated popPK models able to predict AUC of DAB, OHD, and TRA in patients treated with CombiDT. In a context of personalized medicine, the results of our PK/PD analysis conducted on a “real life” cohort of MM patients support the use of plasma DAB monitoring to prevent DLT onset, particularly in fragile patients such as elderly patients. The clinical benefit of plasma OHD monitoring regarding efficacy, and especially the AUC_OHD_/AUC_DAB_ ratio, deserves more investigation in a larger cohort of MM patients.

## Figures and Tables

**Table 1:** Demographic and baseline characteristics of patients.

Characteristics	DAB/OHD Model (*n* = 73)	TRA Model (*n* = 60)
Demographic Data		
Sex, *n* (%)		
Male/Female	43 (59)/30 (41)	33 (55)/27 (45)
Age (years)	61.2 (20.0–90.0)	60.0 (20.0–90.0)
Body weight (kg)	73.0 (51.7–166.0)	73.0 (53.0–166.0)
BMI (kg/m^2^)	25.9 (17.4–44.6)	25.9 (18.3–44.6)
FFM (kg)	53.7 (34.2–94.4)	52.7 (34.7–94.4)
BSA (m^2^)	1.9 (1.0–3.0)	3.1 (2.5–3.0)
Histological tumor type, *n* (%)		
Melanoma	65 (89)	52 (87)
Other ^a^	8 (11)	8 (13)
PPI intake, (*n* %)		
Yes/No	57 (78)/16 (22)	52 (87)/8 (13)
**Baseline Biological Data**		
AST (UI/L)	30.0 (12.0–103.0)	30.0 (12.0–103.0)
ALT (UI/L)	28.0 (10.0–116)	28.0 (13.0–116.0)
Total bilirubin (µmol/L)	6.3 (1.4–47.0)	6.3 (1.4–47.0)
Albumin (g/L)	43.0 (23.0–49.0)	43.0 (31.0–48.0)
CRP (mg/L)	19.9 (1.0–248.0)	17.2 (1.0–248.0)
**Baseline Characteristics of MM Patients (*n* = 52) ^b^**		
8th AJCC stage		
Stage IIIC, *n* (%)	5 (10)	
Stage IV M1a, *n* (%)	8 (15)	
Stage IV M1b, *n* (%)	3 (6)	
Stage IV M1c, *n* (%)	16 (31)	
Stage IV M1d, *n* (%)	20 (38)	
ECOG PS, *n* (%)		
0	29 (56)	
1	16 (31)	
2	5 (9)	
3	2 (4)	
Number of previous treatment lines, *n* (%)		
0	35 (67)	
1	10 (19)	
≥2	7 (14)	
Number of metastatic sites, *n* (%)		
<3/≥3	25 (48)/27 (52)	
Cerebral metastases, *n* (%)		
Yes/No	31 (60)/21 (40)	
LDH, *n* (%)	19.9 (1.0–248.0)	
<1.5 N	39 (75)	
≥1.5 N	13 (25)	

8th AJCC stage, 8th edition of the American Joint Committee on Cancer; ALT, alanine amino transferase; AST, aspartate amino transferase; BMI, body mass index; BSA, body surface area; CRP, C-reactive protein FFM, free-fat mass; ECOG PS, Eastern Cooperative Oncology Group Performance Status; LDH, lactate dehydrogenase; MM, metastatic melanoma; PPI, proton-pump inhibitors. Results are expressed as median (range) or frequency (percent). ^a^ Anaplastic thyroid carcinoma (*n* = 6), non-small-cell lung carcinoma (*n* = 2). ^b^ Baseline characteristics of MM patients included in the pharmacokinetic/pharmacodynamic analysis.

**Table 2:** Parameter estimates of the base and final dabrafenib/hydroxy-dabrafenib pharmacokinetic model and bootstrap results.

Parameters	Base DAB/OHD Model		Final DAB/OHD Model		Bootstrap (*n* = 500 Samples)	
	Estimate	RSE (%)	Estimate	RSE (%)	Median	PI_95%_
CL/F (L/h)	17.7	6.4	19.3	7.5	19.2	16.0–22.4
V_2_/F (L)	39.5	13.1	39.1	13.7	38.3	26.9–52.1
k_a_ (1/h)	1.8 fixed		1.8 fixed		1.8 fixed	
Q/F (L/h)	3.85	23.0	3.40	21.5	3.32	1.59–6.60
V_4_/F (L)	19.6	19.6	18.7	20.1	18.7	12.5–39.8
CL_m_/F (L/h)	22.8	5.8	23.2	5.9	22.9	19.8–25.9
V_3_/F (L)	5.23	26.2	5.11	30.5	4.99	0.385–9.03
Q_m/F_ (L/h)	7.39	23.7	7.21	22.3	7.02	4.42–12.1
V_5_/F (L)	25.7	20.4	27.1	23.9	24.7	16.1–45.8
Tlag (h)	0.50	0.20	0.499	0.2	0.499	0.295–0.578
θ_age/(CL/F)_	-	-	−0.536	28.4	−0.499	−0.827−0.215
θ_age/(CLm/F)_	-	-	−0.589	33.6	−0.543	−0.879−0.168
θ_sex/(CL/F)_	-	-	0.832	6.4	0.829	0.732–0.932
IIV_CL/F_ (%)	21.9	17.2	16.0	21.9	14.4	5.83–20.9
IIV_V2/F_ (%)	43.0	18.1	50.8	20.0	69.4	44.3–88.9
IIV_CLm/F_ (%)	26.8	11.9	24.0	14.4	21.8	15.5–28.5
IIV_V3/F_ (%)	84.0	47.5	47.5	51.5	82.0	15.9–278
IOV (%)	17.2	11.8	17.4	12.0	17.0	13.1–20.3
RUV of DAB (%)	49.2	6.4	48.7	6.6	48.2	41.8–54.6
RUV of OHD (%)	53.1	6.4	53.1	6.3	52.7	46.2–59.1
RUV_corr_ (%)	87.3	11.5	87.0	2.1	87.3	80.4–92.1

PI95%; 95% percentile interval; CL/F, apparent clearance of DAB; CLm/F, apparent clearance of OHD; IIV, interindividual variability defined as coefficient of variation (%); IOV, inter-occasion variability on CL/F defined as coefficient of variation (%); ka, first-order absorption rate constant; Q/F, inter-compartment clearance of DAB; Qm/F, inter-compartment clearance of OHD; RSE, relative standard error defined as SE/estimate, with SE directly retrieved from NONMEM; RUV, residual unexplained variability; RUVcorr, correlation between the proportional error components; Tlag, lag time, V2/F, apparent central volume of distribution of DAB; V3/F, apparent central volume of distribution of OHD; V4/F, apparent peripheral volume of distribution of DAB; V5/F, apparent peripheral volume of distribution of OHD; θage/(CL/F), Age effect on CL/F; θage/(CLm/F), Age effect on CLm/F; θsex/(CL/F), Sex effect on CL/F. Final model equations for individual CL/F and CLm/F: CL_ind/F = CL/F × (1 + θage/(CL/F) × (AGE-MAGE)/MAGE) × (θsex/(CL/F)Sex) × exp(IIVCL/F_ind). CLm_ind/F = CLm/F × (1 + θage/(CLm/F) × (AGE-MAGE)/MAGE) × exp(IIVCLm/F_ind). with MAGE = 61.2 years, median AGE value in the study population; Sex being 0/1 for man/woman, and the rest as previously defined.

**Table 3:** Parameter estimates of the final trametinib (TRA) pharmacokinetic model and bootstrap results.

Parameters	Final TRA Model		Bootstrap (*n* = 500 Samples)	
	Estimate	RSE (%)	Median	PI_95%_
CL/F (L/h)	5.83	4.6	5.82	5.35–6.35
V_2_/F (L)	61.9	26.8	65.1	22.3–125.4
k_a_ (1/h)	0.913	38.2	1.04	0.390–3.10
Q/F (L/h)	64.9	23.4	62.6	33.6–116.8
V_3_/F (L)	417.0	42.2	448.2	193.9–2145.0
Tlag (h)	0.709	17.1	0.728	0.505–1.06
IIV_CL/F_ (%)	29.6	16.3	28.6	18.9–38.0
IIV_Q_ (%)	80.2	23.7	80.1	30.6–167.1
RUV (ng/mL)	4.14	6.2	4.10	3.69–4.61

PI_95%_; 95% percentile interval; CL/F, apparent clearance of TRA; IIV, interindividual variability defined as coefficient of variation (%); k_a_, first order absorption rate constant; Q/F, inter-compartment clearance of TRA; RSE, relative standard error defined as SE/estimate, with SE directly retrieved from NONMEM; RUV, residual unexplained variability (additive error); Tlag, lag time; V_2_/F, apparent central volume of distribution of TRA; V_3_/F, apparent peripheral volume of distribution of TRA.

**Table 4:** Risk factors for dose-limiting toxicity onset in BRAF-mutated metastatic melanoma patients treated with combination of DAB plus TRA (CombiDT).

Parameters	DLT	No DLT	*p* Value
	Dabrafenib/Hydroxy-dabrafenib		
AUC_DAB_ (ng∙h/mL)	9624 (8121–11676)	7485 (3399–17712)	0.0065
AUC_OHD_ (ng∙h/mL)	7509.5 (4918–10300)	5812 (2459–10300)	0.16
AUC_COMPOSITE_ (ng∙h/mL)	16855 (13491–21976)	13605 (5877–28012)	0.030
Age * (years)	54.5 (37–81)	59 (20–90)	0.66
BMI * (kg/m^2^)	25.9 (20.4–33.4)	25.1 (19.6–40.9)	0.87
Sex			0.094
Male	8 (67%)	14 (35%)	
Female	4 (33%)	26 (65%)	
ECOG PS *			0.51
0-1	8 (67%)	21 (52%)	
≥2	4 (33%)	19 (48%)	
LDH			0.47
<1.5N	10 (83%)	27 (68%)	
≥1.5N	2 (17%)	13 (32%)	
	**Trametinib**		
AUC_TRA_ (ng∙h/mL)	268 (144–448)	268 (111–750)	0.47
Age * (years)	55 (37–90)	61 (20–89)	0.61
BMI * (kg/m^2^)	25.9 (20.4–35.5)	25.2 (19.6–40.9)	0.68
Sex			0.37
Male	9 (53)	13 (37)	
Female	8 (47)	22 (63)	
ECOG PS *			1
0–1	10 (59)	19 (54)	
≥2	7 (41)	16 (46)	
LDH			0.33
<1.5N	9 (53)	20 (57)	
≥1.5N	8 (47)	15 (43)	

AUC, area under the plasma concentration over interval administration; BMI, body mass index; DLT, dose-limiting toxicity; ECOG PS, Eastern Cooperative Oncology Group Performans Status; LDH, lactate dehydrogenase. * Baseline parameters. Results are expressed as median [range] or frequency (percent).

**Table 5:** Univariate and multivariate Cox proportional hazard analyses of risk factors for death and progression.

Univariate Model	Risk of Death		Risk of Progression	
	HR (CI_95%_)	*p* Value	HR (CI_95%_)	*p* Value
Sex	1.47 (0.46–4.67)	0.51	1.04 (0.44–2.47)	0.82
Age	1.01 (0.97–1.05)	0.64	0.99 (0.96–1.02)	0.63
BMI	1.01 (0.86–1.18)	0.89	0.99 (0.87–1.12)	0.84
ECOG PS ≥2	6.64 (1.31–33.78)	0.022	1.98 (0.72–5.46)	0.19
Number of metastatic sites ≥3	3.20 (0.86–11.93)	0.083	3.64 (1.27–10.39)	0.016
Cerebral metastases	2.51 (0.79–7.96)	0.12	4.05 (1.47–11.19)	0.0070
PPI	3.51 (0.90–13.71)	0.071	1.80 (0.51–6.38)	0.57
LDH	0.55 (0.12–2.52)	0.44	1.03 (0.36–2.90)	0.96
AUC_M3 DAB_	1.02 (0.75–1.39)	0.88	1.04 (0.82–1.32)	0.75
AUC_M3 OHD_	1.64 (1.09–2.48)	0.019	1.37 (1.02–1.83)	0.037
AUC_M3 Composite_	1.15 (0.93–1.43)	0.20	1.11 (0.94–1.30)	0.21
AUC_M3 ratio OH/DAB_	4.11 (1.18–14.29)	0.026	1.66 (0.62–4.46)	0.31
AUC_M3 TRA_	1.37 (0.94–1.98)	0.10	1.31 (0.93–1.83)	0.12
**Multivariate models**	HR (CI_95%_)	*p* value	HR (CI_95%_)	*p* value
Using AUC_M3,OHD_				
ECOG PS≥2	6.58 (1.29–33.56)	0.023		
AUC_M3,OHD_	1.61 (1.07–2.45)	0.023		
Using AUC_M3,OHD/DAB_				
ECOG PS≥2	16.52 (2.51–108.86)	0.0036		
AUC_M3,OHD/DAB_	10.61 (2.34–48.15)	0.0022		
**Using AUC_M3,OHD_**				
Number of metastasis site ≥3			3.25 (1.11–9.50)	0.032
AUC_M3,OHD_			1.27 (0.97–1.68)	0.088
**Using AUC_M3,OHD_**				
Cerebral metastases			1.23 (1.35–10.39)	0.011
AUC_M3,OHD_			1.29 (0.99–1.68)	0.064

CI_95%_, 95% confidence interval; AUC_M3_ was defined as the mean of AUC estimated during the first 3 months of treatment; AUC_M3_,_OHD/DAB_, ratio of AUC_M3,OHD_/AUC_M3,DAB_ being 0/1 for ratio < 1 vs. ratio ≥ 1 (4th quartile cut off); BMI, body mass index; ECOG PS, Eastern Cooperative Oncology Group Performance Status; HR, hazard ratio; LDH, lactate dehydrogenase; PPI, proton-pump inhibitors being 0/1 for no PPI intake/PPI intake. Results are expressed as median (range) or frequency (percent).

## Supplementary Materials

The following are available online at https://www.mdpi.com/2072-6694/12/4/931/s1, Figure S1: Goodness-of-fit plots of DAB final population pharmacokinetic model, Figure S2: Goodness-of-fit plots of OHD final population pharmacokinetic model, Figure S3: Goodness-of-fit plots of TRA final population pharmacokinetic model.
