# Supplementary material for: Population Pharmacokinetics/Pharmacodynamics of Dabrafenib Plus Trametinib in Patients with BRAF-Mutated Metastatic Melanoma
Source: Cancers (Basel). 2020 Apr 9;12(4):931. doi: 10.3390/cancers12040931 (PMC7226106; doi:10.3390/cancers12040931)
Supplement: Supplementary file 1 [file cancers-12-00931-s001.pdf]

Supplemental Materials: Population Pharmacokinetics/Pharmacodynamics of Dabrafenib Plus Trametinib in Patients with BRAF-Mutated Metastatic Melanoma

David Balakirouchenane, Sarah Guégan, Chantal Csajka, Anne Jouinot, Valentine Heidelberger, Alicja Puszkiel, Ouidad Zehou, Nihel Khoudour, Perrine Courlet, Nora Kramkimel, Coralie Lheure, Nathalie Franck, Olivier Huillard, Jennifer Arrondeau, Michel Vidal, Francois Goldwasser, Eve Maubec, Nicolas Dupin, Selim Aractingi, Monia Guidi and Benoit Blanchet

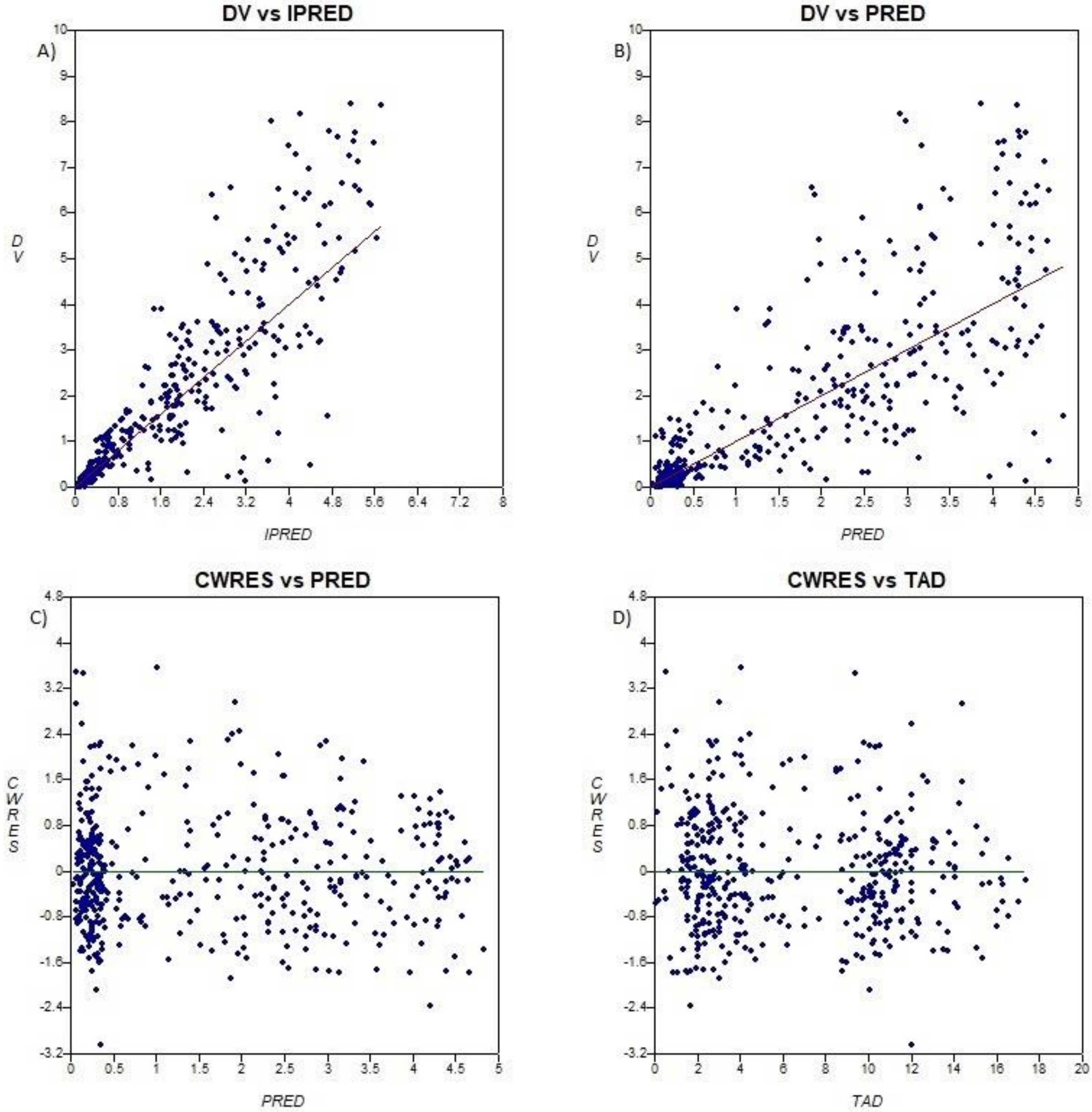

Figure S1. Goodness-of-fit plots of DAB final population pharmacokinetic model. **A)** Observed concentrations (DV, dependent variable) *vs* individual predictions (IPRED); line of identity is printed in red. **B**) Observed concentrations (DV, dependent variable) *vs* population predictions (PRED); identity line is printed in

red. **C**) Conditional weighted residuals (CWRES) *vs* population predictions; ordinate value zero is printed in green. **D**) CWRES *vs* time post-dose; ordinate value zero is printed in green

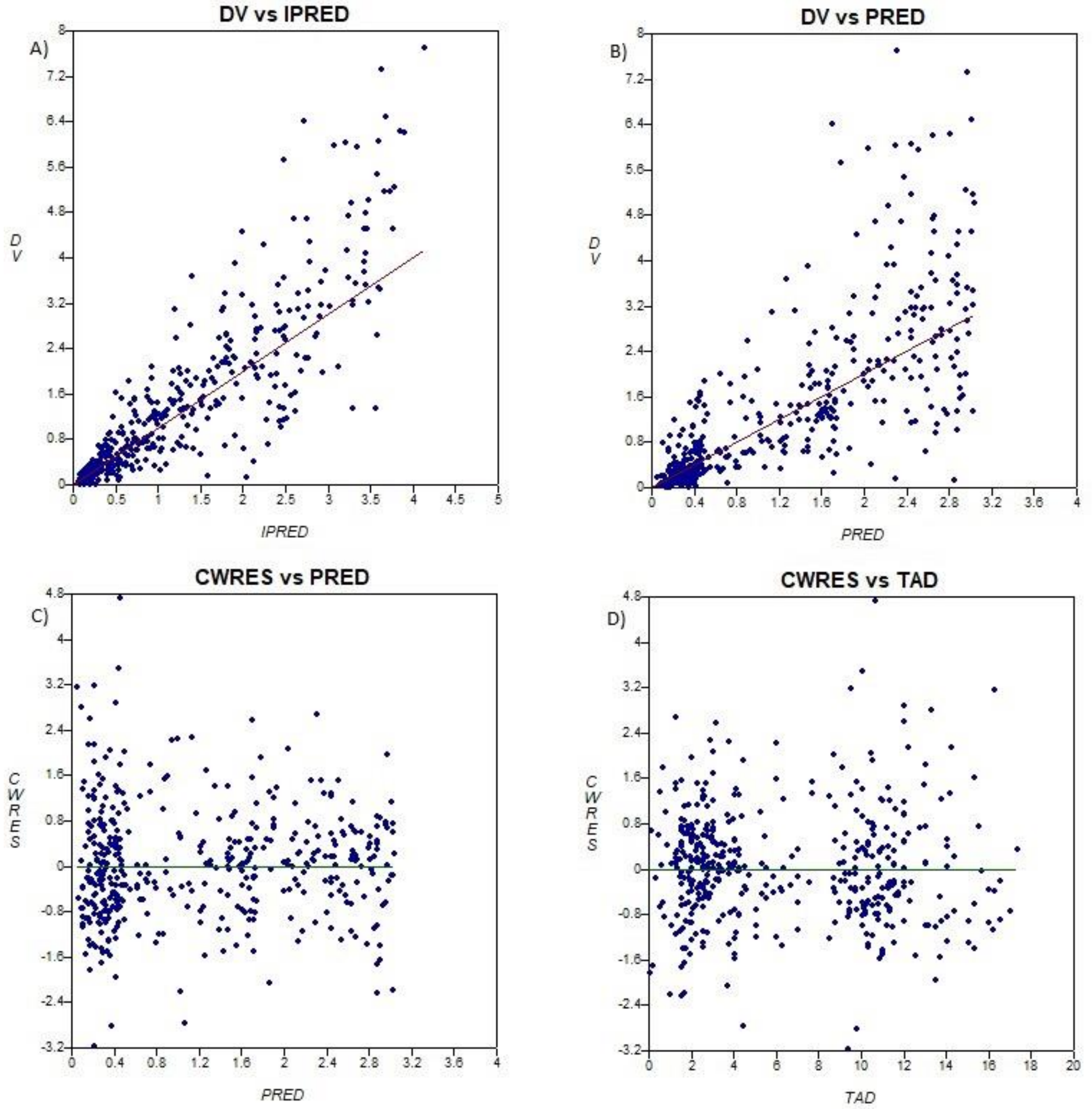

Figure S2. Goodness-of-fit plots of OHD final population pharmacokinetic model. A) Observed concentrations (DV, dependent variable) *vs* individual predictions (IPRED); line of identity is printed in red. **B**) Observed concentrations (DV, dependent variable) *vs* population predictions (PRED); identity line is printed in red. **C**) Conditional weighted residuals (CWRES) *vs* population predictions; ordinate value zero is printed in green. **D**) CWRES *vs* time post-dose; ordinate value zero is printed in green

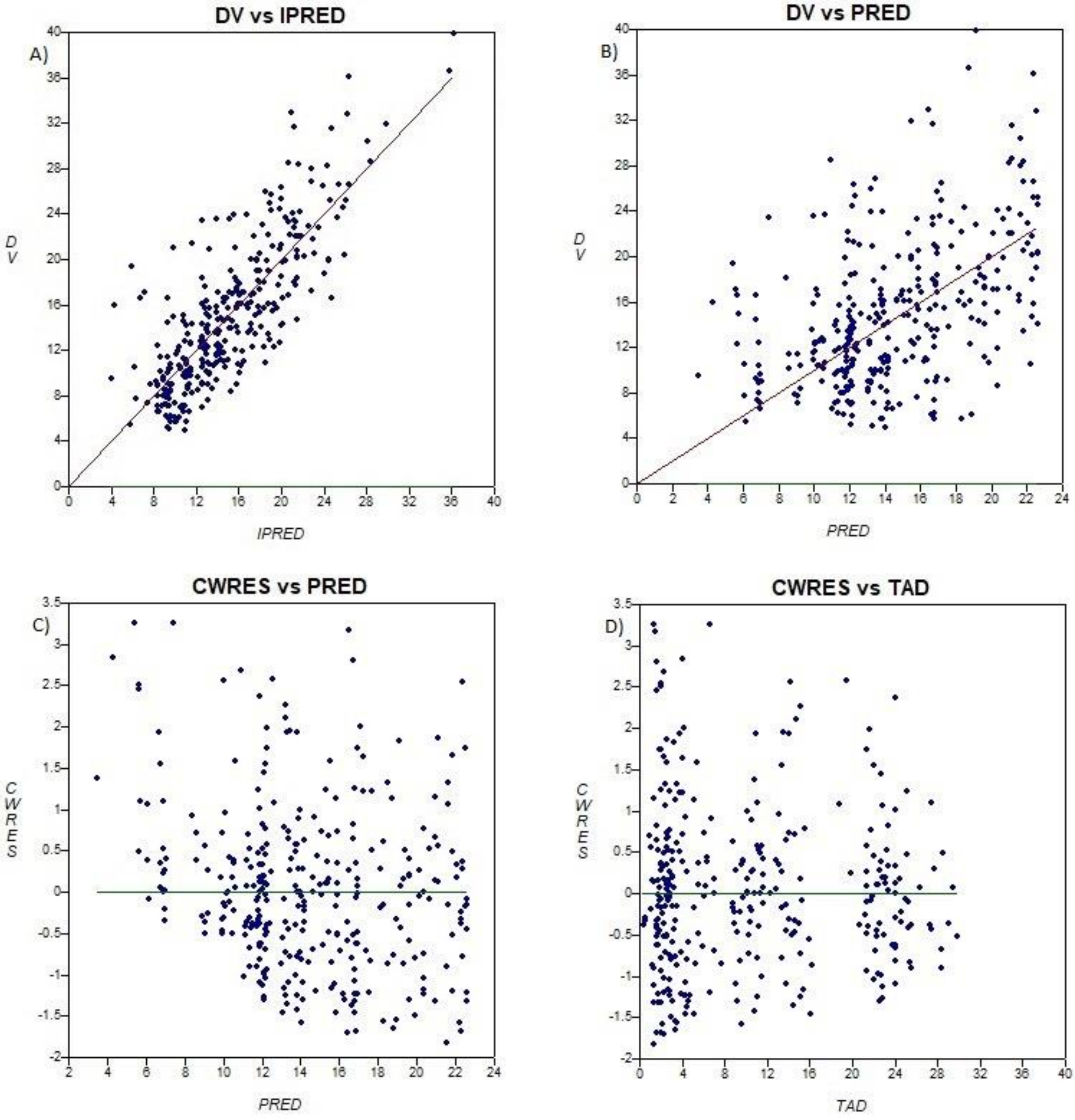

Figure S3. Goodness-of-fit plots of TRA final population pharmacokinetic model. A) bserved concentrations (DV, dependent variable) *vs* individual predictions (IPRED); line of identity is printed in red. **B**) Observed concentrations (DV, dependent variable) *vs* population predictions (PRED); identity line is printed in red. **C**) Conditional weighted residuals (CWRES) *vs* population predictions; ordinate value zero is printed in green. **D**) CWRES *vs* time post-dose; ordinate value zero is printed in green

© 2020 by the authors. Licensee MDPI, Basel, Switzerland. This article is an open access article distributed under the terms and conditions of the Creative Commons Attribution (CC BY) license (http://creativecommons.org/licenses/by/4.0/).
